# 3-Amino-1-methyl-9,10-dihydro­phenanthrene-2,4-dicarbonitrile

**DOI:** 10.1107/S1600536811035008

**Published:** 2011-09-14

**Authors:** Abdulrahman O. Al-Youbi, Abdullah M. Asiri, Hassan M. Faidallah, Khalid A. Alamry, Seik Weng Ng

**Affiliations:** a Chemistry Department, Faculty of Science, King Abdulaziz University, PO Box 80203 Jeddah, Saudi Arabia; b Center of Excellence for Advanced Materials Research, King Abdulaziz University, PO Box 80203 Jeddah, Saudi Arabia; c Department of Chemistry, University of Malaya, 50603 Kuala Lumpur, Malaysia

## Abstract

The asymmetric unit of the title compound, C_17_H_13_N_3_, contains two independent mol­ecules, which are non-planar as they are buckled owing to the ethyl­ene portion. The dihedral angle between the benzene rings is 26.4 (1)° in one mol­ecule and 32.9 (1)° in the other. In the crystal, the mol­ecules are disposed about a false inversion center, and are linked by two N—H⋯N hydrogen bonds, generating a dimer. The dimers are linked by further N—H⋯N hydrogen bonds, resulting in a chain that runs along the longest axis of the ortho­rhom­bic unit cell.

## Related literature

For the synthesis of dihydro­phenanthrenes, see: Dellagreca *et al.* (2000 ▶); Ram & Goel (1997 ▶).
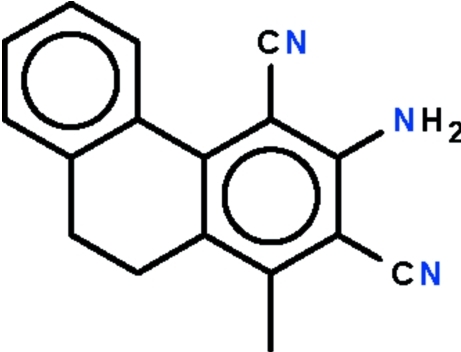


## Experimental

### 

#### Crystal data

C_17_H_13_N_3_
*M* *_r_* = 259.30
Orthorhombic, 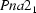

*a* = 26.8587 (7) Å
*b* = 8.8158 (2) Å
*c* = 11.2035 (3) Å
*V* = 2652.78 (12) Å^3^
*Z* = 8
Cu *K*α radiation
μ = 0.62 mm^−1^
*T* = 100 K
0.30 × 0.20 × 0.02 mm

#### Data collection

Agilent SuperNova Dual diffractometer with an Atlas detector
Absorption correction: multi-scan (*CrysAlis PRO*; Agilent, 2010 ▶) *T*_min_ = 0.836, *T*_max_ = 0.988
10819 measured reflections
2800 independent reflections
2621 reflections with *I* > 2σ(*I*)
*R*_int_ = 0.033

#### Refinement

*R*[*F* ^2^ > 2σ(*F* ^2^)] = 0.034
*wR*(*F* ^2^) = 0.091
*S* = 1.09
2800 reflections
379 parameters
1 restraint
H atoms treated by a mixture of independent and constrained refinement
Δρ_max_ = 0.16 e Å^−3^
Δρ_min_ = −0.20 e Å^−3^

### 

Data collection: *CrysAlis PRO* (Agilent, 2010 ▶); cell refinement: *CrysAlis PRO*; data reduction: *CrysAlis PRO*; program(s) used to solve structure: *SHELXS97* (Sheldrick, 2008 ▶); program(s) used to refine structure: *SHELXL97* (Sheldrick, 2008 ▶); molecular graphics: *X-SEED* (Barbour, 2001 ▶); software used to prepare material for publication: *publCIF* (Westrip, 2010 ▶).

## Supplementary Material

Crystal structure: contains datablock(s) global, I. DOI: 10.1107/S1600536811035008/xu5310sup1.cif

Structure factors: contains datablock(s) I. DOI: 10.1107/S1600536811035008/xu5310Isup2.hkl

Additional supplementary materials:  crystallographic information; 3D view; checkCIF report

## Figures and Tables

**Table 1:** Hydrogen-bond geometry (Å, °)

*D*—H⋯*A*	*D*—H	H⋯*A*	*D*⋯*A*	*D*—H⋯*A*
N2—H21⋯N4	0.91 (4)	2.15 (4)	3.007 (3)	156 (3)
N2—H22⋯N6^i^	0.91 (3)	2.38 (3)	3.265 (3)	164 (2)
N5—H51⋯N1^ii^	0.91 (4)	2.12 (4)	3.012 (3)	168 (3)
N5—H52⋯N3	0.91 (3)	2.41 (3)	3.283 (3)	161 (3)

Symmetry codes: (i) 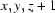; (ii) 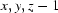.

**Fig. 1.**
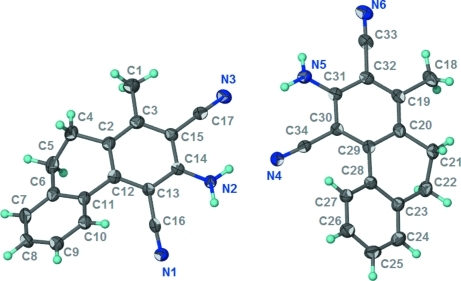
Thermal ellipsoid plot (Barbour, 2001) of C17H13N3 at the 70% probability level; hydrogen atoms are drawn as spheres of arbitrary radius.

## supplementary crystallographic information

### Comment

As the dihydrophenanthrene skeleton is a principal component of a number of pharmaceutical products, there is a large collection of reserach on the synthesis of dihydrophenanthrene compounds. This skeleton is known to mimic natural products, which is yet another source of bioactive compounds (Dellagreca *et al.*, 2000). In this study, we have used 1-tetralone, acetaldehyde, and malonitrile to synthesize the skeleton; an early study reported the use of 1-tetralone to condense with 2*H*-pyran-2-ones to furnish this skeleton (Ram & Goel, 1997). The title molecule, C_17_H_13_N_3_ (Scheme I), is non-planar as the molecule it is buckled owing to the ethylene portion; the dihedral angle between the aromatic rings is 26.4 (1) ° in one independent molecule and 32.9 (1) ° in the other. The molecules are disposed about a false inversion center, and are linked by two *N*–*H*···N hydrogen bonds to generate a dimer (Fig. 1). The dimers are linked by N–H···N hydrogen bonds to result in a linear chain (Table 1).

### Experimental

Acetaldehyde (0.44 g,10 mmol), 1-tetralone (1.46 g, 10 mmol), malononitrile (0.66 g, 10 mmol) and ammonium acetate(6.20 g, 80 mmol) in absolute ethanol (50 ml) were heated for 6 houyrs. The mixture was allowed to cool, and the solid was collected, washed with water, dried and then recrystallized from ethanol to yield light brown crystals, m.p. 457–459 K.

### Refinement

Carbon- and nitrogen-bound H-atoms were placed in calculated positions [C–H 0.95 to 0.99 Å, *U*_iso_(H) 1.2*U*_eq_(C)] and were included in the refinement in the riding model approximation.

The amino H-atoms were located in a difference Fouier map and were freely refined.

The Flack parameter initially refined to 0.1 (4) on 1933 Friedel pairs. As the uncertainty was too large, Friedel pairs were merged.

### Crystal data

**Table:**

C_17_H_13_N_3_	*F*(000) = 1088
*M**_r_* = 259.30	*D*_x_ = 1.299 Mg m^−^^3^
Orthorhombic, *P**n**a*2_1_	Cu *K*α radiation, λ = 1.54184 Å
Hall symbol: P 2c -2n	Cell parameters from 4366 reflections
*a* = 26.8587 (7) Å	θ = 3.3–74.4°
*b* = 8.8158 (2) Å	µ = 0.62 mm^−^^1^
*c* = 11.2035 (3) Å	*T* = 100 K
*V* = 2652.78 (12) Å^3^	Plate, light brown
*Z* = 8	0.30 × 0.20 × 0.02 mm

### Data collection

**Table:**

Agilent SuperNova Dual diffractometer with an Atlas detector	2800 independent reflections
Radiation source: SuperNova (Cu) X-ray Source	2621 reflections with *I* > 2σ(*I*)
Mirror	*R*_int_ = 0.033
Detector resolution: 10.4041 pixels mm^-1^	θ_max_ = 74.6°, θ_min_ = 3.3°
ω scans	*h* = −24→33
Absorption correction: multi-scan (*CrysAlis PRO*; Agilent, 2010)	*k* = −10→10
*T*_min_ = 0.836, *T*_max_ = 0.988	*l* = −13→13
10819 measured reflections	

### Refinement

**Table:**

Refinement on *F*^2^	Primary atom site location: structure-invariant direct methods
Least-squares matrix: full	Secondary atom site location: difference Fourier map
*R*[*F*^2^ > 2σ(*F*^2^)] = 0.034	Hydrogen site location: inferred from neighbouring sites
*wR*(*F*^2^) = 0.091	H atoms treated by a mixture of independent and constrained refinement
*S* = 1.09	*w* = 1/[σ^2^(*F*_o_^2^) + (0.058*P*)^2^ + 0.0761*P*] where *P* = (*F*_o_^2^ + 2*F*_c_^2^)/3
2800 reflections	(Δ/σ)_max_ = 0.001
379 parameters	Δρ_max_ = 0.16 e Å^−^^3^
1 restraint	Δρ_min_ = −0.20 e Å^−^^3^

### Special details

**Table:**

Geometry. All e.s.d.'s (except the e.s.d. in the dihedral angle between two l.s. planes) are estimated using the full covariance matrix. The cell e.s.d.'s are taken into account individually in the estimation of e.s.d.'s in distances, angles and torsion angles; correlations between e.s.d.'s in cell parameters are only used when they are defined by crystal symmetry. An approximate (isotropic) treatment of cell e.s.d.'s is used for estimating e.s.d.'s involving l.s. planes.

### Fractional atomic coordinates and isotropic or equivalent isotropic displacement parameters (Å^2^)

**Table:**

	*x*	*y*	*z*	*U*_iso_*/*U*_eq_	
N1	0.87010 (7)	0.4611 (2)	0.74979 (18)	0.0264 (4)	
N2	0.89781 (7)	0.4949 (2)	0.45228 (19)	0.0211 (4)	
H21	0.9103 (12)	0.469 (4)	0.379 (3)	0.045 (9)*	
H22	0.9152 (11)	0.471 (3)	0.519 (3)	0.031 (8)*	
N3	0.87032 (8)	0.5796 (2)	0.15228 (18)	0.0281 (4)	
N4	0.94098 (7)	0.3192 (2)	0.24687 (18)	0.0256 (4)	
N5	0.94187 (7)	0.4150 (2)	−0.04685 (19)	0.0254 (4)	
H51	0.9239 (12)	0.424 (4)	−0.115 (3)	0.047 (9)*	
H52	0.9272 (11)	0.451 (4)	0.020 (3)	0.037 (8)*	
N6	0.97783 (8)	0.3851 (2)	−0.34610 (18)	0.0289 (4)	
C1	0.76004 (11)	0.7688 (4)	0.2539 (2)	0.0391 (7)	
H1A	0.7507	0.8757	0.2628	0.059*	
H1B	0.7301	0.7078	0.2399	0.059*	
H1C	0.7828	0.7579	0.1861	0.059*	
C2	0.76447 (8)	0.7425 (3)	0.4785 (2)	0.0229 (5)	
C3	0.78542 (8)	0.7153 (3)	0.3660 (2)	0.0248 (5)	
C4	0.71332 (8)	0.8142 (3)	0.4946 (2)	0.0289 (5)	
H4A	0.6941	0.8069	0.4193	0.035*	
H4B	0.7169	0.9228	0.5155	0.035*	
C5	0.68597 (8)	0.7302 (3)	0.5944 (2)	0.0290 (5)	
H5A	0.6527	0.7758	0.6064	0.035*	
H5B	0.6814	0.6226	0.5718	0.035*	
C6	0.71544 (8)	0.7399 (2)	0.7083 (2)	0.0228 (5)	
C7	0.69360 (8)	0.7612 (3)	0.8196 (2)	0.0269 (5)	
H7	0.6584	0.7626	0.8266	0.032*	
C8	0.72290 (9)	0.7805 (3)	0.9206 (2)	0.0292 (5)	
H8	0.7076	0.7929	0.9964	0.035*	
C9	0.77457 (9)	0.7817 (3)	0.9110 (2)	0.0278 (5)	
H9	0.7946	0.7990	0.9796	0.033*	
C10	0.79671 (8)	0.7576 (3)	0.8011 (2)	0.0229 (5)	
H10	0.8320	0.7593	0.7946	0.028*	
C11	0.76781 (8)	0.7309 (2)	0.6998 (2)	0.0206 (4)	
C12	0.78952 (8)	0.6957 (2)	0.5817 (2)	0.0208 (4)	
C13	0.83376 (7)	0.6109 (2)	0.57199 (19)	0.0181 (4)	
C14	0.85599 (7)	0.5797 (2)	0.4602 (2)	0.0183 (4)	
C15	0.83135 (8)	0.6366 (2)	0.3579 (2)	0.0213 (4)	
C16	0.85397 (7)	0.5328 (2)	0.67296 (19)	0.0202 (4)	
C17	0.85254 (8)	0.6055 (3)	0.2431 (2)	0.0230 (5)	
C18	1.06712 (10)	0.1026 (3)	−0.2549 (2)	0.0305 (5)	
H18A	1.1034	0.1134	−0.2536	0.046*	
H18B	1.0535	0.1591	−0.3228	0.046*	
H18C	1.0584	−0.0049	−0.2626	0.046*	
C19	1.04565 (8)	0.1645 (2)	−0.1407 (2)	0.0223 (4)	
C20	1.06385 (8)	0.1173 (2)	−0.0295 (2)	0.0215 (4)	
C21	1.10732 (8)	0.0077 (3)	−0.0178 (2)	0.0259 (5)	
H21A	1.1112	−0.0505	−0.0928	0.031*	
H21B	1.1384	0.0653	−0.0039	0.031*	
C22	1.09836 (9)	−0.1007 (3)	0.0855 (2)	0.0285 (5)	
H22A	1.0694	−0.1661	0.0672	0.034*	
H22B	1.1278	−0.1669	0.0959	0.034*	
C23	1.08876 (7)	−0.0150 (3)	0.1992 (2)	0.0235 (5)	
C24	1.10496 (8)	−0.0667 (3)	0.3099 (2)	0.0293 (5)	
H24	1.1220	−0.1609	0.3151	0.035*	
C25	1.09665 (9)	0.0173 (3)	0.4127 (2)	0.0302 (5)	
H25	1.1064	−0.0217	0.4882	0.036*	
C26	1.07405 (8)	0.1582 (3)	0.4049 (2)	0.0273 (5)	
H26	1.0701	0.2189	0.4744	0.033*	
C27	1.05719 (8)	0.2104 (3)	0.2956 (2)	0.0222 (5)	
H27	1.0418	0.3071	0.2908	0.027*	
C28	1.06245 (8)	0.1233 (2)	0.1924 (2)	0.0208 (4)	
C29	1.04170 (8)	0.1703 (2)	0.0757 (2)	0.0189 (4)	
C30	0.99925 (8)	0.2638 (2)	0.06895 (19)	0.0188 (4)	
C31	0.98132 (8)	0.3191 (2)	−0.0416 (2)	0.0199 (4)	
C32	1.00643 (8)	0.2697 (2)	−0.1454 (2)	0.0213 (4)	
C33	0.99065 (8)	0.3313 (3)	−0.2578 (2)	0.0234 (5)	
C34	0.96871 (8)	0.2955 (2)	0.1702 (2)	0.0200 (4)	

### Atomic displacement parameters (Å^2^)

**Table:**

	*U*^11^	*U*^22^	*U*^33^	*U*^12^	*U*^13^	*U*^23^
N1	0.0289 (9)	0.0307 (10)	0.0196 (9)	0.0063 (8)	−0.0002 (8)	0.0018 (8)
N2	0.0200 (8)	0.0266 (9)	0.0167 (8)	0.0044 (7)	0.0001 (8)	−0.0012 (8)
N3	0.0344 (10)	0.0287 (10)	0.0210 (10)	0.0030 (8)	0.0028 (9)	0.0007 (8)
N4	0.0239 (9)	0.0305 (10)	0.0223 (9)	0.0050 (7)	0.0007 (8)	0.0002 (8)
N5	0.0283 (9)	0.0297 (10)	0.0180 (9)	0.0066 (8)	−0.0039 (9)	−0.0016 (8)
N6	0.0320 (10)	0.0326 (11)	0.0221 (10)	−0.0069 (8)	−0.0006 (9)	0.0013 (9)
C1	0.0415 (14)	0.0554 (17)	0.0203 (12)	0.0196 (13)	−0.0049 (11)	0.0002 (13)
C2	0.0224 (11)	0.0263 (11)	0.0200 (11)	0.0039 (8)	−0.0023 (9)	−0.0020 (9)
C3	0.0270 (11)	0.0280 (11)	0.0194 (11)	0.0037 (9)	−0.0048 (9)	−0.0005 (9)
C4	0.0249 (11)	0.0379 (13)	0.0239 (12)	0.0110 (9)	−0.0044 (9)	−0.0020 (10)
C5	0.0195 (10)	0.0338 (13)	0.0337 (13)	0.0038 (9)	−0.0023 (10)	−0.0070 (11)
C6	0.0213 (10)	0.0196 (10)	0.0275 (12)	0.0009 (8)	0.0009 (9)	−0.0002 (9)
C7	0.0219 (10)	0.0244 (11)	0.0344 (13)	0.0030 (8)	0.0104 (10)	0.0033 (10)
C8	0.0345 (12)	0.0303 (12)	0.0226 (12)	0.0072 (9)	0.0103 (10)	0.0036 (10)
C9	0.0325 (12)	0.0313 (12)	0.0197 (11)	0.0059 (9)	0.0007 (10)	−0.0018 (10)
C10	0.0224 (10)	0.0240 (11)	0.0225 (11)	0.0017 (8)	0.0004 (9)	−0.0014 (9)
C11	0.0200 (10)	0.0188 (10)	0.0229 (11)	0.0016 (8)	0.0024 (9)	−0.0014 (9)
C12	0.0188 (10)	0.0211 (11)	0.0224 (11)	−0.0017 (8)	−0.0019 (9)	−0.0017 (9)
C13	0.0178 (9)	0.0197 (10)	0.0170 (10)	−0.0005 (8)	−0.0008 (8)	−0.0027 (8)
C14	0.0187 (9)	0.0183 (9)	0.0178 (9)	−0.0026 (7)	−0.0008 (8)	−0.0007 (8)
C15	0.0240 (10)	0.0231 (10)	0.0169 (10)	0.0006 (8)	−0.0003 (9)	−0.0017 (9)
C16	0.0173 (9)	0.0236 (10)	0.0195 (11)	−0.0005 (8)	0.0030 (8)	−0.0035 (9)
C17	0.0242 (10)	0.0235 (11)	0.0214 (11)	0.0009 (8)	−0.0027 (9)	0.0018 (9)
C18	0.0410 (13)	0.0276 (12)	0.0229 (11)	−0.0014 (10)	0.0092 (11)	−0.0054 (10)
C19	0.0277 (10)	0.0186 (10)	0.0206 (10)	−0.0034 (8)	0.0050 (9)	−0.0023 (9)
C20	0.0231 (10)	0.0185 (10)	0.0229 (11)	−0.0011 (8)	0.0054 (9)	−0.0007 (9)
C21	0.0260 (10)	0.0216 (10)	0.0299 (12)	0.0040 (8)	0.0078 (9)	−0.0008 (10)
C22	0.0298 (11)	0.0190 (11)	0.0365 (13)	0.0040 (8)	0.0090 (11)	0.0027 (10)
C23	0.0185 (10)	0.0224 (11)	0.0295 (12)	0.0004 (8)	0.0037 (9)	0.0052 (10)
C24	0.0238 (11)	0.0274 (12)	0.0367 (13)	0.0032 (9)	0.0024 (10)	0.0089 (11)
C25	0.0265 (11)	0.0368 (13)	0.0275 (12)	0.0015 (9)	−0.0037 (10)	0.0101 (11)
C26	0.0232 (10)	0.0347 (13)	0.0242 (11)	0.0012 (9)	−0.0015 (9)	0.0027 (10)
C27	0.0180 (9)	0.0252 (11)	0.0235 (11)	0.0002 (8)	0.0002 (8)	0.0034 (9)
C28	0.0196 (9)	0.0194 (10)	0.0235 (11)	−0.0002 (8)	0.0025 (8)	0.0028 (9)
C29	0.0201 (10)	0.0152 (9)	0.0215 (10)	−0.0016 (7)	0.0014 (9)	−0.0004 (8)
C30	0.0202 (10)	0.0181 (10)	0.0180 (11)	−0.0012 (8)	0.0000 (8)	−0.0017 (8)
C31	0.0202 (9)	0.0195 (9)	0.0200 (10)	−0.0028 (7)	−0.0010 (8)	−0.0009 (9)
C32	0.0225 (10)	0.0228 (11)	0.0185 (10)	−0.0054 (8)	0.0000 (9)	0.0007 (9)
C33	0.0243 (10)	0.0244 (11)	0.0215 (11)	−0.0067 (8)	0.0017 (9)	−0.0024 (9)
C34	0.0198 (9)	0.0206 (10)	0.0197 (11)	0.0007 (8)	−0.0033 (9)	0.0015 (8)

### Geometric parameters (Å, °)

**Table:**

N1—C16	1.153 (3)	C12—C13	1.408 (3)
N2—C14	1.352 (3)	C13—C14	1.414 (3)
N2—H21	0.91 (4)	C13—C16	1.431 (3)
N2—H22	0.91 (3)	C14—C15	1.415 (3)
N3—C17	1.147 (3)	C15—C17	1.433 (3)
N4—C34	1.156 (3)	C18—C19	1.506 (3)
N5—C31	1.357 (3)	C18—H18A	0.9800
N5—H51	0.91 (4)	C18—H18B	0.9800
N5—H52	0.91 (3)	C18—H18C	0.9800
N6—C33	1.150 (3)	C19—C20	1.401 (3)
C1—C3	1.505 (3)	C19—C32	1.405 (3)
C1—H1A	0.9800	C20—C29	1.400 (3)
C1—H1B	0.9800	C20—C21	1.521 (3)
C1—H1C	0.9800	C21—C22	1.520 (3)
C2—C12	1.400 (3)	C21—H21A	0.9900
C2—C3	1.400 (3)	C21—H21B	0.9900
C2—C4	1.523 (3)	C22—C23	1.504 (3)
C3—C15	1.419 (3)	C22—H22A	0.9900
C4—C5	1.529 (4)	C22—H22B	0.9900
C4—H4A	0.9900	C23—C24	1.391 (3)
C4—H4B	0.9900	C23—C28	1.411 (3)
C5—C6	1.505 (3)	C24—C25	1.388 (4)
C5—H5A	0.9900	C24—H24	0.9500
C5—H5B	0.9900	C25—C26	1.386 (3)
C6—C7	1.391 (3)	C25—H25	0.9500
C6—C11	1.412 (3)	C26—C27	1.385 (3)
C7—C8	1.388 (4)	C26—H26	0.9500
C7—H7	0.9500	C27—C28	1.394 (3)
C8—C9	1.392 (3)	C27—H27	0.9500
C8—H8	0.9500	C28—C29	1.481 (3)
C9—C10	1.384 (3)	C29—C30	1.409 (3)
C9—H9	0.9500	C30—C31	1.416 (3)
C10—C11	1.395 (3)	C30—C34	1.427 (3)
C10—H10	0.9500	C31—C32	1.413 (3)
C11—C12	1.479 (3)	C32—C33	1.435 (3)
			
C14—N2—H21	120 (2)	C3—C15—C17	119.8 (2)
C14—N2—H22	120.1 (18)	N1—C16—C13	175.4 (2)
H21—N2—H22	119 (2)	N3—C17—C15	178.7 (2)
C31—N5—H51	120 (2)	C19—C18—H18A	109.5
C31—N5—H52	121.3 (19)	C19—C18—H18B	109.5
H51—N5—H52	116 (3)	H18A—C18—H18B	109.5
C3—C1—H1A	109.5	C19—C18—H18C	109.5
C3—C1—H1B	109.5	H18A—C18—H18C	109.5
H1A—C1—H1B	109.5	H18B—C18—H18C	109.5
C3—C1—H1C	109.5	C20—C19—C32	119.4 (2)
H1A—C1—H1C	109.5	C20—C19—C18	121.0 (2)
H1B—C1—H1C	109.5	C32—C19—C18	119.6 (2)
C12—C2—C3	119.95 (19)	C29—C20—C19	120.04 (18)
C12—C2—C4	117.3 (2)	C29—C20—C21	117.8 (2)
C3—C2—C4	122.7 (2)	C19—C20—C21	122.2 (2)
C2—C3—C15	119.4 (2)	C22—C21—C20	110.08 (18)
C2—C3—C1	121.0 (2)	C22—C21—H21A	109.6
C15—C3—C1	119.6 (2)	C20—C21—H21A	109.6
C2—C4—C5	108.60 (19)	C22—C21—H21B	109.6
C2—C4—H4A	110.0	C20—C21—H21B	109.6
C5—C4—H4A	110.0	H21A—C21—H21B	108.2
C2—C4—H4B	110.0	C23—C22—C21	110.84 (19)
C5—C4—H4B	110.0	C23—C22—H22A	109.5
H4A—C4—H4B	108.3	C21—C22—H22A	109.5
C6—C5—C4	109.88 (18)	C23—C22—H22B	109.5
C6—C5—H5A	109.7	C21—C22—H22B	109.5
C4—C5—H5A	109.7	H22A—C22—H22B	108.1
C6—C5—H5B	109.7	C24—C23—C28	119.2 (2)
C4—C5—H5B	109.7	C24—C23—C22	122.5 (2)
H5A—C5—H5B	108.2	C28—C23—C22	118.3 (2)
C7—C6—C11	119.2 (2)	C25—C24—C23	121.0 (2)
C7—C6—C5	123.12 (19)	C25—C24—H24	119.5
C11—C6—C5	117.6 (2)	C23—C24—H24	119.5
C8—C7—C6	120.5 (2)	C26—C25—C24	119.8 (2)
C8—C7—H7	119.7	C26—C25—H25	120.1
C6—C7—H7	119.7	C24—C25—H25	120.1
C7—C8—C9	120.2 (2)	C25—C26—C27	119.8 (2)
C7—C8—H8	119.9	C25—C26—H26	120.1
C9—C8—H8	119.9	C27—C26—H26	120.1
C10—C9—C8	119.7 (2)	C26—C27—C28	121.2 (2)
C10—C9—H9	120.1	C26—C27—H27	119.4
C8—C9—H9	120.1	C28—C27—H27	119.4
C9—C10—C11	120.72 (19)	C27—C28—C23	118.8 (2)
C9—C10—H10	119.6	C27—C28—C29	122.69 (19)
C11—C10—H10	119.6	C23—C28—C29	118.5 (2)
C10—C11—C6	119.3 (2)	C20—C29—C30	119.6 (2)
C10—C11—C12	122.96 (18)	C20—C29—C28	119.34 (18)
C6—C11—C12	117.7 (2)	C30—C29—C28	121.0 (2)
C2—C12—C13	119.9 (2)	C29—C30—C31	121.6 (2)
C2—C12—C11	119.17 (18)	C29—C30—C34	122.5 (2)
C13—C12—C11	120.9 (2)	C31—C30—C34	115.62 (18)
C12—C13—C14	121.84 (19)	N5—C31—C32	122.0 (2)
C12—C13—C16	120.98 (19)	N5—C31—C30	121.2 (2)
C14—C13—C16	116.51 (18)	C32—C31—C30	116.83 (18)
N2—C14—C13	121.1 (2)	C19—C32—C31	122.0 (2)
N2—C14—C15	122.1 (2)	C19—C32—C33	120.3 (2)
C13—C14—C15	116.78 (17)	C31—C32—C33	117.64 (19)
C14—C15—C3	121.9 (2)	N6—C33—C32	177.8 (2)
C14—C15—C17	118.24 (18)	N4—C34—C30	175.0 (2)
			
C12—C2—C3—C15	1.7 (3)	C32—C19—C20—C29	2.5 (3)
C4—C2—C3—C15	−174.7 (2)	C18—C19—C20—C29	−176.8 (2)
C12—C2—C3—C1	−178.1 (2)	C32—C19—C20—C21	−177.9 (2)
C4—C2—C3—C1	5.5 (4)	C18—C19—C20—C21	2.8 (3)
C12—C2—C4—C5	−38.2 (3)	C29—C20—C21—C22	37.7 (3)
C3—C2—C4—C5	138.3 (2)	C19—C20—C21—C22	−141.9 (2)
C2—C4—C5—C6	59.0 (3)	C20—C21—C22—C23	−55.3 (3)
C4—C5—C6—C7	140.4 (2)	C21—C22—C23—C24	−144.8 (2)
C4—C5—C6—C11	−37.8 (3)	C21—C22—C23—C28	35.0 (3)
C11—C6—C7—C8	3.0 (3)	C28—C23—C24—C25	−1.5 (3)
C5—C6—C7—C8	−175.2 (2)	C22—C23—C24—C25	178.2 (2)
C6—C7—C8—C9	1.3 (3)	C23—C24—C25—C26	−3.2 (4)
C7—C8—C9—C10	−2.5 (4)	C24—C25—C26—C27	3.9 (3)
C8—C9—C10—C11	−0.5 (3)	C25—C26—C27—C28	0.1 (3)
C9—C10—C11—C6	4.7 (3)	C26—C27—C28—C23	−4.7 (3)
C9—C10—C11—C12	−176.3 (2)	C26—C27—C28—C29	175.5 (2)
C7—C6—C11—C10	−5.9 (3)	C24—C23—C28—C27	5.4 (3)
C5—C6—C11—C10	172.4 (2)	C22—C23—C28—C27	−174.4 (2)
C7—C6—C11—C12	175.08 (19)	C24—C23—C28—C29	−174.83 (19)
C5—C6—C11—C12	−6.6 (3)	C22—C23—C28—C29	5.4 (3)
C3—C2—C12—C13	−5.0 (3)	C19—C20—C29—C30	3.8 (3)
C4—C2—C12—C13	171.6 (2)	C21—C20—C29—C30	−175.78 (19)
C3—C2—C12—C11	177.5 (2)	C19—C20—C29—C28	−178.0 (2)
C4—C2—C12—C11	−5.9 (3)	C21—C20—C29—C28	2.4 (3)
C10—C11—C12—C2	−148.5 (2)	C27—C28—C29—C20	154.0 (2)
C6—C11—C12—C2	30.5 (3)	C23—C28—C29—C20	−25.7 (3)
C10—C11—C12—C13	34.1 (3)	C27—C28—C29—C30	−27.8 (3)
C6—C11—C12—C13	−147.0 (2)	C23—C28—C29—C30	152.4 (2)
C2—C12—C13—C14	4.5 (3)	C20—C29—C30—C31	−6.8 (3)
C11—C12—C13—C14	−178.06 (19)	C28—C29—C30—C31	175.05 (19)
C2—C12—C13—C16	−165.8 (2)	C20—C29—C30—C34	166.3 (2)
C11—C12—C13—C16	11.7 (3)	C28—C29—C30—C34	−11.8 (3)
C12—C13—C14—N2	−178.4 (2)	C29—C30—C31—N5	−176.8 (2)
C16—C13—C14—N2	−7.7 (3)	C34—C30—C31—N5	9.6 (3)
C12—C13—C14—C15	−0.6 (3)	C29—C30—C31—C32	3.2 (3)
C16—C13—C14—C15	170.03 (19)	C34—C30—C31—C32	−170.37 (19)
N2—C14—C15—C3	175.0 (2)	C20—C19—C32—C31	−6.2 (3)
C13—C14—C15—C3	−2.7 (3)	C18—C19—C32—C31	173.1 (2)
N2—C14—C15—C17	−1.5 (3)	C20—C19—C32—C33	173.6 (2)
C13—C14—C15—C17	−179.2 (2)	C18—C19—C32—C33	−7.2 (3)
C2—C3—C15—C14	2.2 (3)	N5—C31—C32—C19	−176.7 (2)
C1—C3—C15—C14	−178.0 (2)	C30—C31—C32—C19	3.3 (3)
C2—C3—C15—C17	178.7 (2)	N5—C31—C32—C33	3.5 (3)
C1—C3—C15—C17	−1.5 (4)	C30—C31—C32—C33	−176.46 (19)

### Hydrogen-bond geometry (Å, °)

**Table:**

*D*—H···*A*	*D*—H	H···*A*	*D*···*A*	*D*—H···*A*
N2—H21···N4	0.91 (4)	2.15 (4)	3.007 (3)	156 (3)
N2—H22···N6^i^	0.91 (3)	2.38 (3)	3.265 (3)	164 (2)
N5—H51···N1^ii^	0.91 (4)	2.12 (4)	3.012 (3)	168 (3)
N5—H52···N3	0.91 (3)	2.41 (3)	3.283 (3)	161 (3)

Symmetry codes: (i) *x*, *y*, *z*+1; (ii) *x*, *y*, *z*−1.
